# Supplementary material for: Are carbon emissions trading and green financial instruments synergistic? -Comprehensive quantitative research based on content analysis
Source: PLoS One. 2024 Mar 7;19(3):e0298601. doi: 10.1371/journal.pone.0298601 (PMC10919601; doi:10.1371/journal.pone.0298601)
Supplement: S1 Data — (ZIP) [file pone.0298601.s001.zip › Data/地级市Z_F工作报告.zip!/╣π╢1⁄2/╔╟═╖╩╨/└┤╫╘╣1⁄2╓┌║┼í╛┬φ┐╦╩2╛▌═°í┐_2012.docx]

各位代表：

　　现在，我代表汕头市人民政府，向大会报告工作，请予审议，并请各位政协委员和其他列席人员提出意见。

　　一、工作回顾

　　过去五年，全市人民在省委、省政府和市委的正确领导下，坚持以科学发展观统揽全局，全面贯彻落实省粤东工作会议的决策部署，积极应对国际金融危机的严重冲击和各种挑战，经济社会保持平稳较快发展，顺利完成市第十二届人代会提出的主要目标任务。预计2011年全市生产总值1425亿元，同比增长13%，五年间年均增长12.2%；人均生产总值26300元，年均增长10.3%；来源于我市的财政收入216.07亿元，比增26.7%，年均增长22.6%，其中一般预算收入85.58亿元，比增17.8%（按可比口径比增25.1%），年均增长19.6%，经济总体实力跃上新台阶。固定资产投资额420亿元，比增38.4%，年均增长19.2%，出现新一轮投资建设大热潮。进出口总额87亿美元，比增18.7%。港口货物吞吐量突破4000万吨，集装箱吞吐量突破100万标箱，其中对台直航航线开通以来集装箱量年均增长35%，港口发展步入快车道。城镇居民人均可支配收入17400元，农村居民人均纯收入7700元，年均分别增长9.7%、10.5%；城镇登记失业率从5年前的3.1%下降到2.4%；高中阶段教育毛入学率超过85%，民生民安逐步改善。节能减排完成“十一五”目标任务。我市又新增了“国家园林城市”、“中国城市信息化50强”、“国家知识产权工作示范城市”、“国家电子商务示范城市”等城市名片，“国家卫生城市”再次通过复审确认。

　　特别是2010年以来，根据中央和省委对汕头发展的新要求，在中共汕头市委的正确领导下，市政府进一步树立科学发展理念，努力探索科学发展新模式，团结带领全市人民，抢抓特区扩围和发展新机遇，坚定信心，砥砺奋进，成功实现赶超进位，2010年全省落实科学发展观考核排位比2009年上升了3位，经济社会发展呈现新气象。

　　城市发展有新格局。积极谋求汕头在国家层面的发展定位，先后被确定为国家主体功能区重点开发区域、海峡西岸经济区中心城市、国家海洋经济发展试点地区，区域中心城市地位进一步显现。完成市、区、镇三级土地利用规划修编，为拓展城市发展空间提供用地保障。加大城市规划力度，以“多规融合”启动新一轮城市总体规划修编，高标准规划“一核多组团”的城市发展新格局和“一湾两岸”的城市形态，城市规划的战略性、前瞻性不断增强。启动市场运作、资本运营机制，整合盘活城市优质资源，加强与中信、中交等大型央企的战略合作，重点打造濠江新城、东海岸新城、珠港新城和西部生态新城，城市空间结构不断优化。濠江新城首期南滨片区和苏埃湾过海隧道项目奠基启动，东海岸新城已形成陆域面积约7平方公里，珠港新城完成整体规划设计，西部生态新城金凤西路一期工程开工建设。围绕区域交通一体化，积极谋划一批交通基础设施项目，全力推进港口、铁路、高速路网建设，主动与周边城市对接，城市交通功能不断提升。

　　产业发展有新举措。积极推进现代产业体系建设，编制实施战略性新兴产业倍增计划、现代服务业提速计划、传统优势产业提升计划和海洋综合开发计划。积极引进一批世界500强企业和大型央企参与产业发展。出台《现代产业用地使用权出让办法》、《加强总部经济发展的若干意见》等措施，推进产业集聚、高端发展，产业转型升级迈出新步伐。加快建设国家玩具质量监督检验中心（汕头）等一批公共服务平台，促进企业自主创新能力的提升。形成17个产业集群，澄海玩具礼品、龙湖工艺毛织服装、龙湖输配电设备列入省级产业集群升级示范区，成为国家玩具礼品、化妆品、包装装潢印刷产业重要生产基地。鼓励扶持企业上市，现有上市企业19家，名列全省前茅。2011年新登记各类企业3218户，其中124户注册资本超过1000万元，投资创业呈现良好势头。

　　重点领域改革有新突破。创新机制，加强对国有资源的监管整合运营，严肃规范国土、规划管理，整体推进国企改革和资产盘活，成功吸引一批现代产业项目落户发展。大力推动行政管理体制改革创新，启动濠江区行政体制综合配套改革，下放471项管理权限，实行大部制，政府部门由22个整合为14个，率先以特区立法形式明确市、区责权划分和调整程序。加快地方金融改革步伐，汕头商行重组成功；广东澄海潮商村镇银行挂牌开业，成为粤东首家村镇银行；潮阳、澄海、南澳3家农信社完成统一法人产权改革；成立9家小额贷款公司，金融服务体系不断健全，金融生态环境逐步改善。

　　增进民生福祉有新成效。制定出台《加强社会建设的决定》，编制幸福汕头客观指标评价体系和“十二五”民生发展规划，并纳入政绩考核体系，民生发展目标更加明晰。全市财政投入民生领域支出96.24亿元，占一般预算支出的68.3%。开展社会治安综合治理，扎实推进平安汕头建设。设立市、区两级价格调节基金，建立低收入群众临时价格补贴与物价上涨联动机制。教育、文化、医疗卫生等社会事业加快发展。去年确定的十件民生实事按时完成年度任务：提前一年完成省下达的80%以上贫困户脱贫任务，城镇居民基本医疗保险和新型农村合作医疗保险实现全覆盖，年人均收入低于1500元的困难家庭全部纳入低保，新增就业8.3万人，解决农村68.1万人饮水安全问题，实施校安工程项目52个，新增公共汽车307部，增加视频监控点5.9万个，建设保障性住房9169套。

　　依法行政水平有新提升。认真执行市人大及其常委会决议，自觉接受人大的法律监督、工作监督和市人民政协的民主监督。在全国地级市率先出台《行政程序规定》，率先开展行政复议委员会试点工作。进一步优化市行政服务中心服务流程，实施市级建设工程并联审批，配套试行重点（重大）项目全程无偿代办服务制，建立行政电子监察系统，实现省、市、区县、镇街四级联网。按照第四轮行政审批制度改革要求，削减和调整32%的审批事项。启动建设全市统一的行政审批和公共服务综合信息系统，开通网上行政审批大厅，方便企业和群众办事，政务公开和电子政务建设进一步推进。

　　各位代表！过去五年，我们所取得的成绩和进步，是省委、省政府和市委坚强领导的结果，凝聚了全市人民的辛勤劳动。在此，我代表汕头市人民政府，向奋斗在各条战线的全市广大干部群众、海内外乡亲及外地来汕建设者，向给予我们支持的人大代表、政协委员、各民主党派、工商联和社会各界人士，向中央和省驻汕单位，向驻汕解放军、武警官兵，向所有关心和支持汕头发展的港澳台同胞、海外侨胞和国际友人表示崇高的敬意和衷心的感谢！

　　我们也清醒地认识到，与中央和省的要求相比，与人民群众、海内外潮人的期盼相比，我们的工作还有不少问题和不足，经济社会发展还存在不少困难和挑战：一是产业结构调整优化任务艰巨，资源环境约束趋紧，经济发展质量效益不高。二是区域发展不平衡，非中心城区基础设施、环保设施、市政管理、公共服务欠帐较多，促进城乡一体化发展需要付出巨大努力。三是政府历史债务沉重，财税得益率一直偏低，财力薄弱，民生事业投入力不从心，一些民生难题迫切需要解决，相当一部分群众仍存在就业难、住房难、看病难等问题。四是经济管理水平不高，政府资源经营不善，国企改革严重滞后，影响科学发展的体制机制瓶颈依然不少，深层次的改革攻坚任务十分繁重。五是社会管理不到位，交通秩序、治安形势、市容市貌尚未根本改观，群众反映强烈。六是政府职能转变缓慢，有些政府部门工作落实不力，相互间协调配合不够，形式主义、官僚主义不同程度存在；有些公务员公仆意识不强，为纳税人服务观念淡薄，甚至违法违纪、贪污腐败。我们必须直面人民群众的期待，直面自身工作中存在的问题，勇于担当，坚决改进，切实解决。

　　二、今后五年的目标任务

　　各位代表，前不久，我们隆重庆祝汕头经济特区建立三十周年，胜利召开中共汕头市第十次代表大会，确定了建设幸福汕头的重大发展战略。今后五年，对汕头的发展至关重要。我们欣喜地看到，汕头经济特区的发展再一次上升为国家战略，我市正迎来新一轮大发展的重大历史机遇；不少重大发展项目紧锣密鼓、全面地推进，为汕头的发展提供强大的动力；更加可喜的是，全市上下人人思发展、个个想振兴，社会各界求新求快求变的愿望十分强烈，干事创业的氛围日渐浓厚。尽管世界经济环境更为严峻复杂，国内经济发展中不平衡、不协调、不可持续问题仍很突出，区域竞争日趋激烈，我市在前进道路上仍面临诸多挑战，但只要我们振奋精神，坚定信心，自加压力，敢于担当，锐意改革创新，奋力攻坚克难，就一定能够推进汕头经济社会跨越发展，就一定能够夺取建设幸福汕头的丰硕成果。

　　今后五年，我市经济社会发展的主要奋斗目标是：在全面完成“十二五”规划的基础上，到2016年，全市生产总值达到4000亿元，年均增长20%左右；人均生产总值达到71000元，年均增长19%左右；地方财政一般预算收入达到280亿元，年均增长26%，力争经济社会发展总体上赶超全省平均水平，全面实现“十年大发展”目标。

　　实现上述目标，市政府必须奋力推进市第十次党代会提出的建设幸福汕头十大战略举措的落实，突出做到“四个更加注重”：

　　更加注重擦亮特区牌子，全力深化改革开放。要立足新起点，把握新机遇，充分发挥国家层面赋予汕头的政策和定位优势，以敢闯敢试、敢为人先的精神，积极推进体制机制创新，建设法治、阳光、服务政府，以体制机制大转型为科学发展破题探路。坚持与时俱进、海纳百川，以更加开放包容的心态，创造一个公开公平公正的投资创业环境，凝聚海内外各种资源，形成推动汕头跨越式发展的强大合力。

　　更加注重统筹城乡发展，全力加快城乡一体化步伐。要按照“一核多组团”的大特区城市发展新格局，整合全市规划资源，优化提升城市功能，加快推进城乡规划布局、产业发展、市场体制、基础设施、公共服务、管理体制一体化，促进城乡均衡发展、协调发展、可持续发展，不断增强区域中心城市的辐射带动功能。

　　更加注重保障和改善民生，全力提高人民群众的幸福感。要坚持以人为本、执政为民，按照基本公共服务均等化的要求，加大民生投入，顺应民情，纾解民困，全面提高居民收入、公共服务和社会福利水平，努力使全市人民学有所教、劳有所得、病有所医、老有所养、住有所居，实现社会公平正义，提高人民群众的幸福感。

　　更加注重构筑现代产业体系，全力夯实幸福汕头的物质基础。要深入贯彻落实科学发展观，按照集约化、内涵式发展的新要求，以加快建设现代产业体系为着力点，推动产业结构调整和转型升级，优化产业空间布局，全力推动招商引资和产业培育，催生一批产值超百亿元的行业龙头企业，打造一批产值超千亿元的产业集群和产业园区，促进产业规模与效益同步提升。

　　我们有决心有信心，经过五年的艰苦奋斗，把汕头建设成为实践科学发展、深化改革开放、各种发展活力竞相迸发、各种发展资源充分涌流的创新型特区，成为城乡统筹协调发展、服务辐射能力增强、生活舒适安全、滨海生态怡人、潮汕特色彰显、时代气息浓厚的区域中心城市，为加快建设经济殷实、精神富足、文化多彩、环境优美、民主法治、社会和谐、宜居宜业的幸福汕头打下具有决定性意义的基础。

　　三、2012年工作安排

　　今年政府工作的总体要求是：认真落实省委十届十一次全会、市第十次党代会精神，以科学发展为主题，以建设幸福汕头为目标，擦亮特区牌子，深化改革开放，全面统筹城乡发展，加快推进大特区城市建设和产业转型升级，加强社会管理创新，切实保障和改善民生，推动经济社会发展迈上新台阶。

　　今年经济社会发展的预期目标是：地区生产总值增长15%，工业增加值增长15.4%，第三产业增加值增长15.5%，地方财政一般预算收入增长18%，工业总产值增长15.4%，农业总产值增长3.5%，社会消费品零售总额增长20%，外贸出口总额增长16%，外贸进口总额增长18%，固定资产投资额增长30%，实际吸收外商投资总额增长25%，人口自然增长率控制在7.5％。以内，城镇登记失业率控制在2.5%以内，居民消费价格指数涨幅控制在4%左右，城镇居民人均可支配收入增长14.5%，农村居民人均纯收入增长15%。节能减排指标完成省下达的控制目标任务。

　　实现全年目标任务，各级政府及各职能部门要充分发挥主观能动性，认真履行职责，积极创先争优，在此基础上，集中力量重点抓好六方面工作：

　　（一）加快构建区域中心城市现代综合交通体系，提升集聚辐射服务功能

　　完善发达的交通基础设施，对汕头实现跨越式发展至关重要。在全国各地交通大发展的黄金时期，汕头没有与时俱进、抓住机遇，未能形成畅通的对内对外互联成网的立体交通网络，特别是深水港建设滞后，严重制约了经济的快速发展。基于此，从今年开始，必须集中资源、集中力量攻坚克难，加快启动对内对外通道建设，完善现代综合交通运输体系，进一步增强城市集聚辐射和服务功能，让更多的人流、物流、资金流、信息流汇集，在服务和辐射周边中提升区域中心城市的地位。

　　港口方面。加快促成中远集团等大型码头运营商、航运龙头企业全面参与汕头港基础设施建设，整合港口资源，发展物流业务及船舶运输服务业务。启动广澳港区10万吨级集装箱码头、2万吨级石化码头、海门港区等一批重点项目的建设和项目核准。全力推进广澳港区防波堤工程建设，推动粤东煤炭中转基地尽快正式营运。加大力度优化大通关环境，保持进出口稳定增长，培育提升汕头港作为区域性大宗物资和原材料进口港的地位。做强做大对台直航航线。

　　高速公路和干线公路方面。要根据特区扩围和区域中心城市的新要求，围绕对接潮汕机场和厦深高铁等重大交通枢纽，着力做好交通规划，加快完成汕头至揭阳高速公路（机场路）汕头段建设，确保2013年全线完工；开工建设汕头至揭西、潮州至惠州、揭阳至惠来高速公路汕头段，完成潮汕二环高速公路工程可行性研究前期工作，并力争列入省“十二五”立项开工项目，实现汕潮揭公路互联成网，为推进区域一体化、同城化创造条件。加速完成省道潮汕路路面大修，启动国道206线大学路段改造工程，更新提升通往新机场的快速通道，实现与潮汕机场的高效对接。完成国道324线澄海路段、和惠路段大修工程，金凤路桥黄河路段改造、陈沙公路西延段工程；启动国道324线潮阳潮南路段改造、省道司神线陈沙公路至惠来段路面大修工程、泰山北路跨梅溪河桥建设。力争南澳大桥年底合龙建成。

　　铁路方面。全力配合厦深高铁汕头段建设，争取铁路部门尽早启动厦深高铁进汕头站联络线工程建设。加快推进汕头港疏港铁路、阜鹰汕铁路梅州至汕头段前期工作。积极配合省编制粤东城际轨道交通网络规划。

　　（二）强化产业引导扶持和规范管理，促进产业集聚发展转型升级

　　推进产业集聚发展和转型升级，是实现集约化、内涵式发展的必由之路，是夯实幸福汕头物质基础的迫切需要。要加强产业规划引导和政策扶持，加大招商引资力度，充分发挥侨乡、海峡西岸和特区开放优势，策划、包装、推介一批重大产业合作项目，吸引国内外大中型企业、本地民资和海内外潮商“三种资源”投资落户汕头，同时，扶持本土企业做大做强、做专做精，推动产业集聚发展、转型升级。

　　加强产业规划和园区建设。实施《南部沿海产业带产业布局调整优化的意见》和《工业产业发展布局规划》，明确各类园区的产业定位布局，严格产业准入，突出主导产业，推动形成布局合理、特点鲜明、配套完善的产业集群。研究出台鼓励企业进园区集聚的政策措施，在园区准入、土地供应、费用减免、融资、财政扶持等方面加大力度，引导企业向园区集中。通过园区配套共建、资源共享、污染共治，降低企业投资成本，完善产业链条。充分发挥高新区在政策鼓励、科技服务平台建设上的优势，重点扶持一批科技创新型项目扩大规模，发挥效益。加快创建综合保税区，推动保税区与广澳港港区联动。抓住入选省十大产业转移重点园区的契机，全力加快濠江电子电路工业园、南山湾科技园、潮南产业转移工业园、广澳片、海门片园区基础设施建设，重点招引高端制造业和高新技术产业项目进园发展，推动形成新的经济增长极。整合现有工业园区土地资源，完善园区基础设施配套，抓好园区二次开发。

　　提升传统优势产业。认真贯彻落实国家和省《鼓励进口产品和技术目录》进口贴息政策，支持企业扩大先进技术、关键装备的进口。加快国家精细化工质量监督检验中心（汕头）、中国检验检疫科学研究院（粤东）玩具安全研究中心、国家家用纺织品安全检测重点实验室、国家玩具检测重点实验室、广东出口玩具与礼品公共技术服务平台、粤东云计算中心等的建设，为传统产业改造提升搭建公共服务平台。创造政产学研合作发展条件，促进政府、企业与大型央企、高等院校、科研机构建立技术联盟、产业联盟和标准联盟，推进科研成果产业化。加强农业科技创新，加快发展都市精品农业，提高农业综合素质和效益。积极推进遍布各地的传统建筑业转型升级，打造全国“建筑之乡”。

　　加快发展现代服务业。结合城市总体规划和产业规划，利用“三旧改造”政策，加速推进城区“优二进三”，鼓励采取土地置换、土地使用权调整、改变土地用途等方式盘活存量建设用地，有效拓展现代服务业发展空间。着力改造提升传统商贸、餐饮等传统服务业，充分利用中心城区商贸流通业发达、高端商业写字楼集聚的优势，进一步加强商业网点规划布局，加快推进百脑汇、苏宁电器、红星美凯龙、星湖城、龙湖乐园、华润万家、长江路北、广厦正大广场等一批城市商业综合体建设，增强大城市商业集聚辐射功能，推动综合百货超市、文化娱乐购物中心等高端服务业态迅速发展。实施《加强总部经济发展的若干意见》，启动珠港新城总部园区建设，优先安排上市企业总部进驻。着力发展现代金融服务业，积极引进金融机构，支持鼓励金融改革创新，加快发展村镇银行、创业（风险）投资、小额贷款、融资担保等各类机构，促进金融与实体经济的良性互动发展。大力推动企业改制上市，争取新增上市企业4家以上。加快澄海国际玩具商贸物流城、广澳物流园区、粤东物流新城、谷饶国际针织内衣商贸城、峡山现代物流中心建设，筹划建设区域性进口商品交易中心。大力推进“国家电子商务示范城市”建设，启动“汕头数据特区”项目。

　　培育发展战略性新兴产业。加快出台促进战略性新兴产业发展的政策措施，在土地保障、资金引导、项目建设、技术攻关、市场拓展、人才支持、政府服务等方面加大扶持，破解制约新兴产业发展的难题。统筹安排中央、省、市的扶持资金，采用竞争性扶持形式，重点扶持战略性新兴产业、装备制造业、文化创意产业做大做强。强化投资引导，重点发展新能源、新材料等新兴产业。推动重点企业与央企或海内外大型企业战略合作，启动建设“中国锆城”东里片区、汕头卫星应用产业园区、电容式触摸屏产业等项目。加快推进合成云母产业化基地、数字化超声仪器生产基地、益德环保新型包装材料、西北航空科技飞机刹车片、鑫瑞新型防伪包装材料、猛狮纯电动汽车研发等项目建设。

　　实施海洋旅游综合开发。充分发挥海湾和海岛生态资源优势，规划国际邮轮码头、游艇基地、帆板基地、直升机场和文化、体育比赛集训场所，开发无居民海岛，发展海上运动、海岛冬季阳光旅游等旅游项目，引进战略合作伙伴，争取把南澳打造成为集休闲、商务、会展、观光、度假为一体的高端滨海休闲旅游度假岛。加快开发南山湾、北山湾、海门湾，引进品牌酒店，打造高端休闲度假区。

　　规范市场主体经营管理。坚持“以清理促规范，以规范促发展”的原则，持续不断地开展经济户口清理，强化各类市场主体经营管理，依法规范所有经营商户生产经营证照，严肃税收征管，推行“阳光税务”，落实税负公平，引导企业自觉承担社会义务和社会责任，共同营造共赢可持续发展环境。加强部门间信息共享，推进征信体系建设。在规范清理中强化企业安全生产主体责任，加大对生产、仓储、生活“三合一”单位安全生产的专项整治，有效防止恶性安全事故发生。加大力度打击制假售假违法行为，建立健全打假长效机制。进一步推广清洁生产技术，加快高效节能产品的研发和应用，扎实推进节能减排工作。

　　（三）全力推进新型城市化建设管理，推动城乡区域协调发展

　　加强城乡统筹，树立城市转型发展新理念，走符合汕头实际的文明、宜居、承载力和可持续发展能力强的城市化道路，打造粤东城市群，提升综合竞争力，是汕头人民的共同期盼，也是建设幸福汕头的内在要求。

　　树立现代化城市建设发展理念，加快推进大特区城市建设。要以绿色、智慧、包容、人本的现代城市建设发展理念，进一步强化规划对统筹配置空间资源的基础性、先导性和规范性作用，全面开展覆盖城乡规划编制工作，不断推动城市化布局、形态、风貌有新突破。要深化与战略投资者的合作，加速推进“一湾两岸”城市核心圈建设，精心打造“一核多组团”的大特区城市新格局。濠江新城项目要加快南滨片区12平方公里土地开发和基础设施建设，开工建设苏埃湾过海隧道。珠港新城要抓紧完成控制性详细规划编制，整合盘活土地资源，尽快启动珠港新城总部经济园区建设。东海岸新城完成围内土地吹填约15平方公里，完成新津、新溪、塔岗围三个片区海堤抛石工程，启动市政基础设施配套建设。西部生态新城要抓紧引入战略合作伙伴，发挥依托汕头大学和连接潮汕机场、厦深高铁站的优势，共同规划打造以高新产业、文化产业、现代服务业为重点的生态低碳智慧新城，今年要加快金凤西路一期工程，尽快启动金凤西路二期项目建设和土地储备运营开发。加快潮阳东部新城、潮南行政新区、厦深高铁潮阳站（谷饶）物流新城、澄海“中国锆城”等一批项目的战略合作开发。同时，按照省委、省政府的要求，牵头推动汕潮揭同城化发展，近期以便民惠民为切入点，先行推进交通基础设施建设、车辆通行便利化、电视信号互相落地、电话同网同费、医疗卫生协作等合作事项。

　　创新城乡发展投融资机制，推动城乡综合运营开发。发挥政府资金引导作用，充分利用各种金融杠杆和市场融资功能，改革创新城市投融资建设运营体制，建立可持续发展的城乡建设投融资机制和平台，着力解决城乡建设、城市更新的资金瓶颈。开放市政公用事业、城乡基础设施建设市场和运营市场，积极引进战略投资者，实施综合运营开发。加强监管和搞活并举，加快国企改革步伐，整合盘活国有资产和资源。加强土地管理，大力清理闲置土地，完善土地储备管理经营机制，实现土地有效盘活和节约高效利用。争取第一季度完成发行城投债一期13亿元，精心策划一批条件成熟、带有全局性、利于滚动开发、良性循环的城市开发项目，最大限度发挥资金使用效率。

　　加强城市综合管理，力促城市面貌有新改观。提高城市管理法制化、精细化和信息化水平，建立城市管理长效机制。按照责权利相统一的原则，构建专业管理相对集中、综合管理重心下移、符合市情的网格化管理模式，提升管理效能，增强防灾减灾应急能力。从今年开始，每年实施一批市政基础设施整饰美化工程和街区形象提升工程，完善城市标识系统，推进样板路、严管路、文明路建设，确保一年见成效、三年大变样。积极推动西片旧城区整体策划、规划和改造，传承和挖掘城市历史文化内涵，保护彰显历史文化特色街区。认真处置历史遗留问题，在保护中改造小公园历史文化街区。启动老港区改造，建设融合邮轮码头、潮汕文化展示、亲水休闲、保留历史印记的潮人码头历史文化公园。推进一批破损道路、“水浸街”等基础设施更新改造，逐步改变西片区城市破旧面貌，改善城市主出入口形象。科学规划市区交通路网，梳理优化道路“微循环”，重点改造市区主要交通拥堵点，打通一批断头路和瓶颈路，规划建设一批公交车停靠站，分时段分路段实施不同的交通流组织形式，缓解中心城区交通拥堵现状。持之以恒开展交通秩序综合整治，大力推进机关警力下沉，提高路面“见警率”和“管事率”，严肃查处各种交通违法行为，坚决取缔无牌无证、假牌假证机动车辆，严格出租车营运管理。坚持公交优先发展战略，发展绿色低碳公交，加快提升城市公交服务水平，为广大人民群众提供便捷、安全、舒适的公交服务。

　　实施城乡环境卫生整治工程，着力改善生态环境。汕头依山傍海，生态良好，宜居环境一直是我们为之自豪的城市名片，但是，公共基础设施建设滞后，环保投入不足，生活垃圾、工业废水正在偷偷侵蚀这一幅如画美景。特别是广大农村环境卫生，事关汕头的形象、农民的幸福感。没有农村环境卫生的整洁，就没有幸福农村，也就不可能建成幸福汕头。今年要在全市1064个村（居）全面开展千村环境卫生整治行动，重点支持农村和涉农社区整治垃圾脏乱问题，提高农村绿化美化水平，逐步改善农村生产、生活环境。同时加快名镇名村示范村建设，每年创建2个名镇、市级名村和示范村（社区）。加快推进绿道网和生态景观林带建设。引导发展循环经济，全力推动贵屿废弃电器电子产品集中处理，加快首期500亩园区建设，争取尽快成为国家级现代循环经济产业园。加强城乡环境保护和监控执法力度，积极推进环保电厂、污水处理厂及配套管网建设，提高城镇生活垃圾无害化处理率及污水收集率。加大印染、造纸、电镀等高污染行业的专项整治力度，争取国家、省和社会资本共同开展练江流域综合整治。大力推进农田水利基础设施建设，完成汕头大围等一批重点水利工程。

　　（四）创新社会管理和公共服务，增强人民群众的安全感幸福感

　　建设幸福汕头，核心就是提高人民群众的幸福感。必须回归政府职责本位，积极回应群众的期待和诉求，大力加强以民生为重点的社会管理和公共服务，着力解决就业、教育、社会保障、住房、安全、社会治安等民生问题，全力增强人民群众的安全感幸福感。

　　采取切实举措维护社会和谐稳定，提高人民群众安全感。要牢记宗旨，敢于直面和妥善解决长期积累的深层次矛盾，积极完善畅通群众依法表达诉求的有效渠道，以最大诚意、最大决心及时解决好群众合理、合法利益诉求。同时，坚持法律为上，彰扬法治，坚决维护经济社会发展安定环境。要加快社会治安视频监控系统建设，构建全方位的城乡一体化社会治安防控体系，实现对全市社会治安的多层次、全方位、全时空有效覆盖。深入开展打黑除恶专项行动，做到“黑恶必除，除恶务尽”。加大打击路面“双抢”力度，确保发案率下降10%以上。加大食品药品安全专项整治力度，规范市场秩序，逐步建立长效监管机制，切实保障人民群众的饮食用药安全。全面落实安全生产“一岗双责”，深化安全生产检查整治活动，严厉打击非法违法生产经营建设行为专项行动，切实减少各类事故总量，严防较大以上事故发生。

　　着力解决群众饮用水问题。水资源是城市发展战略资源，必须全方位加强水环境治理和保护。强化韩江流域联防联治，加大饮用水源水质监控监测力度，严厉查处和打击河道非法采砂、堤围乱搭建乱开垦行为。优化潮阳引韩供水工程方案，创新投融资方式，上半年全面启动工程建设，切实办好这项十年前省委、省政府确定的民心工程。确保南澳引韩供水工程竣工投产。启动村村通自来水工作，改建扩建新建一批水厂，整合供水管网，建设应急供水工程，彻底扭转农村“靠天喝水”的被动局面，提高供水保证率和供水安全性。全面完成中心城区“城中村”和涉农社区供水直抄到户改造任务，推进多层住宅小区二次供水改造。创新水务投融资平台，重组涉水国有优质资产，赋予其开展水务投融资和建设、运营、管理职能，形成政府主导、企业参与、市场化运作的投融资模式。改革城乡水务监管体制机制，改善农村供水经营管理方式，强力整治层层转包、加价等现象。

　　完善多层次的社会保障体系。按照“大社保、大经办、大服务”和“惠民、利民、便民”的要求，大力整合各种社会保障资源，加快推进城乡一体化社会保障制度建设，全面发放城乡居民人手一张的社会保障卡，实现城乡居民全覆盖的基本保障。从今年开始，每年安排50个岗位，通过政府购买服务的方式，将服务期满、考核合格的大学生村官择优选聘到街道（镇）从事社会管理和公共服务工作，提高基层服务水平。进一步完善城乡一体化基本医疗保障制度，引入商业保险公司参与居民补充医疗保险，居民可选择按30元和120元两个档次参加基本医疗保险，年度最高可报金额分别达到10万元和12万元，力争参保率达到95%以上。建立城乡一体化居民养老保险制度，力争参保率达到60%。保持对粮油肉菜等重点农产品价格的密切监测，做好价格异动预案，规范市场价格秩序，推动新一轮“菜篮子”工程建设，加强重要生活物资储备，确保市场供给。完善城乡低保标准与价格上涨联动机制和低保标准动态调整机制，提高低保补助水平。完善社会福利和慈善体系，大力发展养老服务事业，整合资源，鼓励社会资金开办各类养老服务机构，着力推广居家养老服务示范点，打造社区养老服务精品。建立高龄老人津贴制度，80周岁以上低保、五保老人按每人每月30元的标准发放高龄补（津）贴。继续实施社会救助，加大对孤儿、残障儿童、贫困精神病患者等特殊群体救助力度。创新保障房建设的投入机制、分配管理制度和规划建设机制，探索建立符合汕头实际的公租房制度。进一步推进殡改工作，逐步实施普惠型殡葬惠民政策。

　　切实改善医疗卫生公共服务。围绕全市医疗资源均衡布局，尽快在西片区启动汕头大学医学院附属肿瘤医院易地重建，在南滨片区启动市中医院南区分院建设，在城市东区规划一家综合医院。中心医院门急诊医技综合楼投入使用。加强现有医疗卫生服务资源的合理整合，加大基层卫生投入力度，完善基层医疗卫生服务网络，健全医疗卫生机构城市对口支援基层制度，扩建3家区级人民医院，改造5家镇卫生院，易地重建4家社区卫生服务中心。动员社会资源发展医疗服务，特别是在医疗资源相对缺乏的区域兴办医疗机构，增加医疗机构床位供给。深化医疗卫生体制改革，全面实施国家基本药物制度，积极推进公立医院改革试点。加强人口计生服务，稳定低生育水平。

　　加大投入推进教育事业均衡发展。加快学前教育事业发展，全面实施学前教育三年行动计划，70%农村乡镇建成普惠性规范化中心幼儿园。推动义务教育均衡发展，加大对农村、革命老区、贫困山区和薄弱学校的扶持和政策倾斜，加快义务教育规范化学校建设步伐，力争全市超过60%的义务教育学校达到规范化标准。建成市特殊教育学校新校区。全面提高普及高中阶段教育的整体水平和社会效应，突出发展中等职业技术教育，完成市滨职校、卫生学校、高级技工学校和金平区、潮南区职教中心新校区建设，全面完成省下达的44所普通高中提升办学水平项目任务，全市高中阶段教育毛入学率冲击90%的目标。加强教师队伍建设，建立革命老区、贫困山区农村小学教师生活补贴制度，继续做好特级教师送教下乡工作，优化农村师资队伍素质。加强学校安全工作，落实校车整治和校园安保措施，切实保障广大师生的生命和财产安全。

　　加快推进文化强市建设。加大财政投入，支持成立文化发展基金，鼓励和吸引社会资金投入公益性文化项目，形成多渠道、多层次的文化投入格局。深入实施文化惠民工程和文化设施达标工程，广泛开展群众性文化体育活动，丰富群众精神文化生活。尽快完成市博物馆新馆配套，早日正式开放。加快发展文化创意、新闻出版、广播影视等文化产业。加强文化遗产保护，修缮一批红色纪念馆。深入发掘特区文化、华侨文化、海洋文化和潮汕传统文化的丰富内涵，大力弘扬开放兼容的人文精神。

　　（五）扎实办好十大民生实事，让广大群众得到真正的实惠

　　1、实施千村环境卫生整治。市财政投入1.75亿元，重点支持澄海、潮阳、潮南、南澳和中心城区涉农社区共863个村对生活垃圾脏乱、生活污水乱排放、农村畜禽污物污染、农村厕所卫生设施进行全面整治，重点是建立垃圾收集运输系统，完善垃圾收集清运设施和建设区县生活垃圾集中处理场所，逐步改善农村生态环境，提高农村宜居水平，提升农民幸福感。

　　2、发放贫困地区农村小学教师生活补贴。对全市非中心城区253所革命老区、贫困山区农村小学的在编教师按每人每月300元的标准发放生活补贴，进一步稳定贫困边远地区教师队伍。

　　3、缓解群众出行难、停车难。一是投放100台小型公共汽车，解决小区间和其他道路条件限制等公交服务不足问题，进一步缓解群众出行难；二是实现中心城区一票制公交车出行“一卡通”，为市民乘坐公交车提供分类优惠；三是科学规划、调整和增设中心城区道路停车泊位，全面清理停车设施挪用、占用现象，尽快规划建设一批专业停车场所，缓解群众停车难。

　　4、推进稳价惠民项目建设。新建50家平价商店和一批蔬菜大棚、冷藏设施，保障生产和市场供应，保持农副产品价格基本稳定；适时启动低收入群众临时价格补贴与价格上涨联动机制，发放临时价格补贴，保障低收入群众的基本生活。

　　5、建立城乡一体化社会保障制度。一是对全市60周岁以上未定期领取退休养老金的居民，按每人每月55元的标准发放基础养老金。二是完成全市“一卡通”社会保障卡50%的发放量，2013年底前基本完成发放。三是建设69个街道（镇）基层社会保障服务平台，为群众提供方便、快捷的社会保障服务。

　　6、免费开放中心城区公园。从2012年春节起，中心城区所有公园免收门票，对群众开放。

　　7、全面启动城区燃气管网建设。完善燃气管道及配套建设，提高我市管道燃气普及率，方便市民安全使用燃气。

　　8、全力推进潮阳引韩供水工程建设，启动村村通自来水工程。支持农村社区铺开村村通自来水工程，重点扶持竞争获得省示范区建设的区县。

　　9、建设一批文体设施。在全市建设11个综合文化站、338个行政村（社区）文化室；免费开放中泰立交桥下5个篮球场、2个网球场，在南滨路游泳跳水馆西侧建设10个标准篮球场和4个小型足球场，并向群众免费开放。

　　10、继续推进城乡保障性住房建设。筹建4710套城镇保障性住房，改造农村低收入困难群众危房2711户，逐步解决群众住房难问题。

　　（六）提高行政效能和政府执行力，打造阳光法治服务政府

　　打造公开透明、高效廉洁的政务环境，是建设幸福汕头的重要保障。各级政府及其职能部门必须自觉接受人大及其常委会的法律监督和政协的民主监督，主动听取各民主党派、人民团体和社会各界的意见建议，问需于民，问计于民，问政于民。要以转变政府职能，规范权力运行和行政行为为突破口，推进体制机制创新，加快电子政务建设，努力打造阳光政府、法治政府、服务政府，进一步改善提升投资创业软环境。

　　推动行政管理创新，继续深化行政体制综合配套改革。各地区各部门都要敢于创新，创先争优，力求每个地区、每个部门至少都有一项工作成为全省乃至全国的先进典型。用好用足特区立法权，加大特区立法力度，为体制机制创新提供有力的法制保障。将大部制机构改革、政府职能转变、创新工作流程和管理方式有机结合起来，全方位推进行政管理体制改革，努力建立一个与市场经济发展相适应、与城乡一体化发展相协调的行政管理体制和工作机制。强化街道（镇）社会管理职能，创新城乡社区管理方式，开展城区基层管理扁平化改革试点，进一步整合街道（镇）、社区行政资源，推进社会管理和公共服务重心下移，努力在基层构建“大服务”、“大公共事业”、“大综合执法”、“大综治维稳”工作格局，逐步实现城乡基层公共服务便民、均等、高效。

　　大力简政放权，积极培育和发展社会组织。扎实推进“富县强镇”事权改革，积极推动行政审批事项下放或委托工作，进一步扩大区县、镇级政府行政事务管理和处置权限，增强基层经济社会发展活力。编制政府向社会组织转移职能目录，建立公益事业招投标制度，逐步将事务性、服务性和部分社会管理工作委托相关社会组织承担，促进政府职能转变。改革社会组织登记管理体制，按照宽进严管的原则，放开工商经济类、公益慈善类、社会服务类、群众生活类社会组织的登记注册。按照“一事一法”的方式，开展特区立法，先行启动公益慈善类、社会服务类社会组织立法，推行“去行政化”和“去垄断化”改革，落实优惠扶持政策，鼓励企业、民间自发成立社会组织，形成新的社会服务主体。鼓励支持社会组织从事养老助残、慈善帮困、法律捐助、医卫文体等与民生密切相关的社会服务，构建与市场经济相适应的社会治理结构。

　　打造优良的政务环境，形成廉洁高效、干事创业良好政风。开展“治贪、治庸、治懒、治散”专项整治行动，严肃问责制度，提升执行力，切实改进工作作风。推动《行政程序规定》贯彻实施，对市直各部门和区县政府主要负责人进行系统培训，提高落实《规定》的自觉性。抓紧制订贯彻落实《规定》的配套措施，进一步规范行政程序，保障和监督行政机关程序正当公正。高效行使行政职权，保护公民、法人和其他组织的合法权益。完善网上行政审批大厅审批事项和服务功能，建立健全电子政务的长效管理机制和运行机制，启动市行政审批和公共服务综合信息系统应用，优化行政流程，提高行政效率，规范部门自由裁量权，杜绝权力寻租空间，塑造高效廉洁、公正为民的服务政府。

　　各位代表，汕头跨越式发展的大特区时代已经来临，经济特区新一轮建设征程已经扬帆启航。新时期赋予我们特区人的使命艰巨而光荣。让我们高举中国特色社会主义伟大旗帜，深入贯彻落实科学发展观，凝聚海内外潮人的智慧和力量，在市委的坚强领导下，团结带领全市人民，解放思想，改革创新，勇立潮头，开拓进取，为潮汕大地的振兴崛起，为建设幸福汕头而努力奋斗！
